# Periodontal status of HIV-infected patients undergoing antiretroviral therapy compared to HIV-therapy naive patients: a case control study

**DOI:** 10.1186/2047-783X-17-2

**Published:** 2012-01-30

**Authors:** Ulrich Fricke, Werner Geurtsen, Ingmar Staufenbiel, Alexander Rahman

**Affiliations:** 1 Department of Conservative Dentistry, Periodontology and Preventive Dentistry, Hannover Medical School, Carl-Neuberg-Strasse 1, Hannover 30625, Germany

**Keywords:** HIV, HAART, PBS, periodontitis, PSI

## Abstract

**Background:**

Although severe oral opportunistic infections decreased with the implementation of highly active antiretroviral therapy, periodontitis is still a commonly described problem in patients infected with human immunodeficiency virus (HIV). The objective of the present investigation was to determine possible differences in periodontal parameters between antiretroviral treated and untreated patients.

**Methods:**

The study population comprised 80 patients infected with HIV divided into two groups. The first group was receiving antiretroviral therapy while the second group was therapy naive. The following parameters were examined: probing pocket depth, gingival recession, clinical attachment level, papilla bleeding score, periodontal screening index and the index for decayed, missed and filled teeth. A questionnaire concerning oral hygiene, dental care and smoking habits was filled out by the patients.

**Results:**

There were no significant differences regarding the periodontal parameters between the groups except in the clinical marker for inflammation, the papilla bleeding score, which was twice as high (*P *< 0.0001) in the antiretroviral untreated group (0.58 ± 0.40 versus 1.02 ± 0.59). The participants of this investigation generally showed a prevalence of periodontitis comparable to that in healthy subjects. The results of the questionnaire were comparable between the two groups.

**Conclusion:**

There is no indication for advanced periodontal damage in HIV-infected versus non-infected patients in comparable age groups. Due to their immunodeficiency, HIV-infected patients should be monitored closely to prevent irreversible periodontal damage. Periodontal monitoring and early therapy is recommended independent of an indication for highly active antiretroviral therapy.

## Introduction

The therapy of patients infected with the human immunodeficiency virus (HIV) dates back to 1986, when the monotherapy, azidothymidine, was introduced. At that time, oral manifestations of the immune deficiency syndrome were used, alongside other diagnostic parameters, as markers for disease progression. Thus, oral candidiasis correlated with a progressive immune deficiency, resulting in Candida esophagitis, defines the progression to acquired immunodeficiency syndrome (AIDS; Center for Disease Control Classification System; Category C).

In 1983, 13 years after detection of the virus, an effective long-term therapy was established by combining up to three antiretroviral substances. After the implementation of this highly active antiretroviral therapy (HAART) in 1996 the morbidity and mortality of HIV-infected patients decreased dramatically. The number of opportunistic infections, like oral Kaposi's sarcoma, oral candidiasis, necrotizing periodontal diseases and hairy leukoplakia, also decreased [1,2]. However, these infections are still evidence of immunological damage in treated and untreated HIV-positive patients. Necrotizing periodontal diseases especially may indicate a CD4+ cell count below 200 cells/μL [3]. To reduce the risk of opportunistic infections, including oral manifestations, international medical associations developed guidelines for starting an antiretroviral treatment. For non-symptomatic patients, these guidelines recommend starting treatment when the patient has a CD4+ cell count below 350 cells/μL, depending on cofactors such as viral load, age and clinical progression [4].

For a dentist involved in the care of an HIV patient, recurrent mucosal and/or gingival infections, for example, human papilloma virus, as well as gingival inflammatory processes and necrotizing ulcerative periodontitis, still remain a problem. The quantitative restoration of the immune system and an increase of the CD4+ cell count under HAART do not always correlate with a qualitative obtainment of immunological skills. The degree of immunological damage and the potential for recovery under therapy are not always predictable. Under these conditions, oral lesions (such as candidiasis) are still a marker for immunological incompetence. Nevertheless, the risk of developing severe oral opportunistic infections decreases with a sufficient antiretroviral therapy [1,5].

Most studies and investigations concerning the prevalence and therapy of periodontal diseases in HIV-infection go back to the pre-HAART era or end shortly after this period. Subsequent studies and investigations evaluated either blood parameters like cytokine expression in HIV-infected patients [6] or compared the periodontal status of HIV-positive with HIV-negative patients [7]. Inflammatory markers, particularly cytokine levels, have become more relevant in periodontal diagnosis and treatment. HIV-positive patients show higher levels of interleukin-18 and interleukin-2 expression than patients with healthy periodontal conditions, as well as higher grades of oral inflammation. It has been documented that HIV-positive patients have more severe grades of periodontitis compared with HIV-negative patients with periodontitis [6]. Until now, there were no studies available that compared the dental and periodontal state between treated and untreated HIV-infected patients. Therefore, the aim of this investigation was to investigate if there are any differences regarding the prevalence and severity of periodontal disease between untreated HIV-infected patients and patients under treatment with a viral load under the limit of detection of 40 cop/mL.

## Materials and methods

### Sample

The study protocol was approved by the Ethical Committee of Hannover Medical School (No. 4201) and performed with the understanding and written consent of each subject. The study population comprised 80 HIV-infected patients (7 women and 73 men) between 21 and 60 years of age (mean 41.7 years ± 7.2). The participants were divided into two groups: patients undergoing HAART (HAART group, n = 40) and antiretroviral untreated patients (naïve group, n = 40).

Inclusion criteria for patients were full age (18 years or older); a stable antiretroviral treatment (HAART) with a viral load below limit of detection (40 cop/mL) for the last six months in the HAART-group; and a CD4+ cell count over 350 cells/μL and no antiretroviral treatment in the naive-group (following international guidelines). Exclusion criteria included pregnancy; diabetes mellitus; immunosuppressed patients; and a detectable viral load in the HAART-group.

### Clinical examination

The patients were either examined in the Department of Conservative Dentistry, Periodontology and Preventive Dentistry of the Hannover Medical School or in a private specialized practice (Georgstrasse, Hannover, Germany). All clinical examinations were carried out by a single experienced dentist using the World Health Organization (WHO) periodontal probe (WHO 2002) to ensure consistency of measurements. A periodontal examination was performed, which included a number of parameters (see Table 1 for full definitions). The probing pocket depth (PPD) and gingival recession (GR) were recorded at four sites per tooth (mesio-buccal, disto-buccal, disto-oral and mesio-oral). The clinical attachment level (CAL) was calculated as the sum of PPD and GR. The papilla bleeding score (PBS) is defined as the occurrence of bleeding after a gentle approximal insertion of a periodontal probe. A PBS score of 0 to 5 is determined on all papillae anterior to the second molars and omits readings on the buccal and lingual gingival margins. The periodontal screening index (PSI) is a WHO accepted detection system for periodontal disease and is subdivided into five codes. Code 0 shows healthy circumstances, codes 1 and 2 indicate gingivitis, and codes 3 and 4 indicate periodontitis (see Table 1 for full definitions). Finally, the DMF-T index describes the total count of decayed missed and filled teeth except the third molars.

**Table 1:** Periodontal and dental parameters.

Parameter	Description
**PPD**	Distance between the gingiva and the bottom of the periodontal sulcus, expressed in millimeters

**GR**	Distance between the gingiva and enamel-cemetum-junction, expressed in millimeters

**CAL**	Addition of PPD and GR

**PBS**	Classified in increasing order of inflammation (grade 0-5), based on the strength of bleeding after gentle probing of the gingiva
	0 = healthy gingiva; no bleeding
	1 = edematous, reddened gingiva; no bleeding
	2 = bleeding without flow
	3 = bleeding with flow along gingival margin
	4 = copious bleeding
	5 = severe inflammation, marked redness and edema, tendency for spontaneous bleeding

**PSI**	Divided into five codes and estimates the clinical parameters of plaque, bleeding and pocket depth.
	Code 0 = colored area of the probe remains visible in all the pockets of the sextant. No calculus and no defective margins on restorations. No bleeding is evident on probing.
	Code 1 = similar to Code 0, but bleeding is detected on probing.
	Code 2 = calculus, above or below the marginal gingiva, is detected. Also used to indicate defective restorative margins. Colored area of the probe is still completely visible.
	Code 3 = colored area of the probe is only partly visible in at least one pocket of the sextant indicating a PPD between 3.5 mm and 5.5 mm.
	Code 4 = colored area of the probe is not visible in at least one pocket of the sextant, indicating a PPD more than 5.5 mm.

**DMF-T**	Total count of teeth which are decayed, missing and filled (except third molars)

CAL: clinical attachment level; DMF-T: decayed-missed-filled-teeth; GR: gingival recession; PBS: papilla bleeding score; PPD: probing pocket depth; PSI: periodontal screening index.

Information concerning demographic features, general health, HIV infection history and immunological status were obtained from an anamnestic questionnaire and patients' medical records. Furthermore, the patients filled out a questionnaire regarding their smoking habits, any drug abuse and oral hygiene.

### Statistical analysis

Power and sample sizes were calculated using nQuery Advisor 6.0. (Statistical Solutions, Saugas, MA, USA). A power calculation revealed that our sample size of 80 had 80% power. Documentation and analysis of the data was performed with the data processing program SPSS/PC Version 17.0. for Windows (SPSS Inc., Chicago, IL, USA). The statistical unit for all tests was each individual subject; median and range values were calculated for all parameters. A non parametric test (Mann-Whitney-Test) with a significance level of *P *< 0.05 was used to analyze the differences between the two groups. The analysis of the patients' questionnaires was performed with a Chi-square test.

## Results

Baseline data showed a viral load of 50,636 ± 68,382 cop/mL in the naïve group and < 40 cop/mL in the HAART-group. The CD4+ cell count was 500.5 ± 241.4 cells/μL in the naïve group and 627.5 ± 316.3 cells/μL in the HAART group.

The demographic data showed a mean age of 44 ± 6.12 years with a duration of infection of 14 ± 6.31 years in the HAART group versus a mean age of 36 ± 8.48 years and a duration of infection of 6 ± 4.24 years in the naïve group. There were no significant differences (*P *> 0.05) in age or CD4+ cell count between the groups, while the duration of infection showed a *P-*value of 0.001. For the HAART group, data concerning drug classes, therapy regimen, nadir and duration of HAART were analyzed. The results are shown in Table 2. Their medical histories showed that 15% (n = 6) of the treated group had co-infection with hepatitis C. There were no patients with a hepatitis C co-infection in the naïve group.

**Table 2:** Antiretroviral parameters (HAART group).

Parameter		Percentage of group
**Drug classes**	NRTI + PI	52.5% (n = 21)
	NRTI + NNRTI	27.5% (n = 11)
	NRTI + NNRTI + PI	5.0% (n = 2)
	PI	2.5% (n = 1)
	NRTI	12.5% (n = 5)

**Therapy regimen**	First line ART	68.3%
	Second/third line therapy	31.7%

**Nadir (CD4+)**	275.6 ± 141.3 cells/μL	
**Duration of HAART**	9.2 ± 4.3 years	

ART: antiretroviral therapy; HAART: highly active antiretroviral therapy; NNRTI: non-nucleoside reverse-transcriptase inhibitors; NRTI: nucleoside analog reverse-transcriptase inhibitors; PI: protease inhibitor.

Their smoking habits showed no significant difference between the groups (HAART group, 61% smokers and 39% non-smokers; naive-group, 65.4% smokers and 34.6% non-smokers). Smoking had a significant influence on the parameter CAL (*P *= 0.039) independently of the group, but did not influence any other measured periodontal parameters.

The results of the questionnaire showed no significant difference in oral hygiene behavior between the groups (Table 3).

**Table 3:** Oral hygiene behavior, concerning technique, frequency and duration.

	HAART group (%)	Naïve group (%)
**Toothbrush**		
Manually	51.2	57.5
Mechanically	29.3	27.5
Manually and mechanically	19.5	15.0
**Frequency**		
Once daily or less	14.6	27.5
Twice daily	70.7	65.0
More than twice daily	14.7	7.5
**Duration**		
< 1 minute	22	26,5
2 minutes	46.3	53.5
3 minutes or more	31.7	20

**Figure 1:**
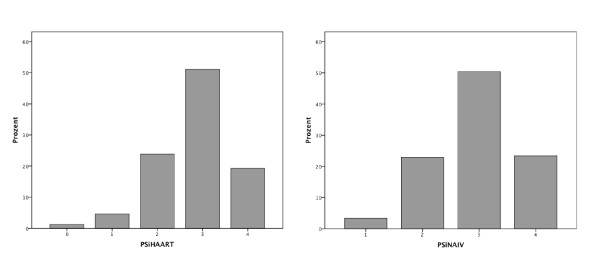
**Percentage occurrence of periodontitis based on the periodontal screening index coding**.

As shown in Figure 1, periodontitis (PSI codes 3 and 4) was diagnosed in 70.2% of the HAART group and 73.7% of the naive-group.

**Figure 2:**
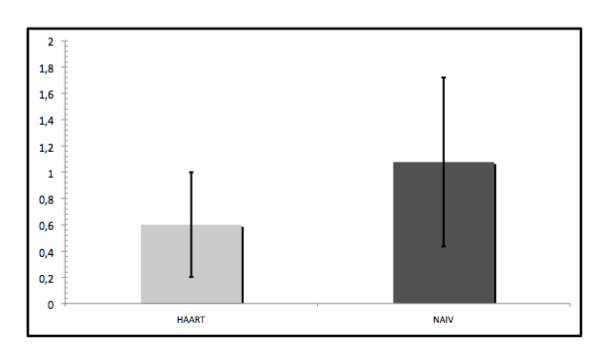
**Mean values and standard deviation of the papilla bleeding score**.

There were no significant differences between the groups regarding PSI (HAART group = 2.7 ± 0.84; naïve group = 2.9 ± 0.70), CAL (HAART group = 3.6 ± 1.96 mm; naïve group = 3.2 ± 1.7 mm) or DMF-T (HAART group = 16.46 ± 6.63; naïve group = 14.57 ± 5.75). As shown in Figure 2, the values for PBS were significantly higher (*P *< 0.0001) in the naïve group (1.02 ± 0.59) compared with the HAART group (0.58 ± 0.40).

## Discussion

The aim of this investigation was to investigate if there are differences in the periodontal status of HIV-infected patients with and without antiretroviral treatment. Previous studies have primarily compared HIV-infected patients with uninfected patients. Our results show a significantly higher level of gingival inflammation in the antiretroviral untreated group of patients although these patients were younger and had a shorter duration of HIV infection. No other examined periodontal parameters showed any significant difference. The demographic data showed an inhomogeneous distribution of gender in both groups. This difference is reflected by the epidemiological data of the Robert Koch Institute and results from the high-risk group, of men having sex with men, that represents the majority of HIV-infected patients in industrialized countries [8]. The difference of patients' age and duration of infection between the groups is consistent with data of other studies, where the median age of starting an antiretroviral treatment was 36 years [9].

For comparable results with HIV-negative patients, a third group of 40 people should have been included to guarantee consistent methods of clinical examination. However, the 2006 4th German Oral Health Study is an appropriate study to use to match our patients with healthy subjects [10]. That study examined the oral health status of different age groups, and their group of 35 to 45 years of age is comparable with the present analysis. Of their examined participants, 73.2% showed a moderate to severe periodontitis (PSI codes 3 and 4), with a DMF-T ranging between 12.9 and 15.6 [10]. These similar results of the present and cited study are evidence for decreasing oral manifestation and revised periodontal conditions in HIV-infected patients. However, a prevalence of 73.2% illustrates the importance of adequate periodontal monitoring and early effective periodontal therapy. The almost similar prevalence between healthy and HIV-infected subjects should not hide the fact that not only clinical parameters like PSI and individual dental care are predictors for the status and progression of periodontal disease in HIV-infected patients. Immunological competence is a marker for success or failure of the antiretroviral therapy as well as the risk of acquiring opportunistic infections. A dentist should closely observe patients on second and third line therapy where an immunological failure has already occurred.

Alongside these clinical results, all patients filled out a questionnaire regarding the frequency and duration of their oral hygiene as well as their smoking habits.

About two-thirds of the patients were smokers, which is described as a main risk factor for periodontal diseases. This study also documents that smoking is an enhancer for attachment loss of periodontal tissues. As a matter of fact, smoking is associated with periodontitis [11] and the risk of suffering from moderate to severe chronic periodontitis is two to eight times higher in people who smoke compared with non-smoking individuals [11-13].

Table 3 shows no significant differences in the oral hygiene behavior between the groups. The results are based on the answers of the patients. The self-reported frequency and duration of brushing are subjective and might be positively influenced by generally accepted guidelines and recommendations. Therefore it can be speculated that the information provided by the patients might be biased.

Most of the published studies regarding HIV and periodontitis in the last 10 years differ between HIV-positive and -negative patients [14-16] or refer to the prevalence of oral opportunistic infections in underdeveloped countries [17-19]. Presently and in the future there will be more patients in industrialized countries who did not experience the pre-HAART era and should therefore not suffer from any opportunistic infections, especially from oral and irreversible periodontal diseases. The examined group under HAART in this investigation showed a mean period of HIV-infection of 14 years. Based on the implementation of HAART in the year 1996, the occurrence of oral adverse events has already decreased.

This investigation primarily focused on clinical parameters of gingival and/or periodontal variation in the examined groups. Further studies regarding laboratory parameters, particularly inflammatory markers and their influence on periodontal diseases in HIV-infected patients with and without antiretroviral treatment, are needed to give a more detailed explanation for the results of this investigation.

## Conclusion

The value for the PBS was almost twice as high in the naïve group compared with the HAART group. Despite the fact that the naïve group had a shorter mean period of HIV-infection and a lower mean age, it showed a higher level of periodontal inflammation, while the progression of periodontal damage was similar in both groups. An explanation for this higher grade of inflammation can only partially be given and needs further investigation to correlate clinical and immunological data in treated and untreated HIV-infected patients and their periodontal status. It may be concluded that HAART seems to avoid progressive periodontal damage even in subjects with a longer period of HIV-infection.

Comparing the periodontal conditions of HIV-positive patients in the present study with a HIV-negative German population (DMS IV [10]), there is no difference in severity and prevalence of periodontal diseases. Smoking has a strong influence on the progression of periodontal disease independent of the immunological status.

A periodontal diagnosis and therapy is recommended independent of an indication for HAART in both groups. The high prevalence of periodontitis strongly suggests the need for prevention, early diagnosis and adequate therapy of periodontitis.

## Competing interests

The authors declare that they have no competing interests.

## Authors' contributions

UF participated in the design of the study, and carried out the clinical examination, patients' interviews and statistical analysis. WG participated in the design of the study and writing of the manuscript. IS participated in the statistical analysis. AR participated in the design and coordination of the study. All authors read and approved the final manuscript.
